# Combined Brain-Perfusion SPECT and EEG Measurements Suggest Distinct Strategies for Speech Comprehension in CI Users With Higher and Lower Performance

**DOI:** 10.3389/fnins.2020.00787

**Published:** 2020-08-11

**Authors:** Mariella Kessler, Irina Schierholz, Martin Mamach, Florian Wilke, Anja Hahne, Andreas Büchner, Lilli Geworski, Frank M. Bengel, Pascale Sandmann, Georg Berding

**Affiliations:** ^1^Department of Nuclear Medicine, Hannover Medical School, Hanover, Germany; ^2^Cluster of Excellence Hearing4all, Hannover Medical School, University of Oldenburg, Oldenburg, Germany; ^3^Department of Otorhinolaryngology, Hannover Medical School, Hanover, Germany; ^4^Department of Otorhinolaryngology, University of Cologne, Cologne, Germany; ^5^Department of Medical Physics and Radiation Protection, Hannover Medical School, Hanover, Germany; ^6^Department of Otorhinolaryngology, Faculty of Medicine Carl Gustav Carus, Saxonian Cochlear Implant Center, Technical University Dresden, Dresden, Germany

**Keywords:** single-photon emission computed tomography, electroencephalography, cochlear-implant, memory, N400, speech processing

## Abstract

Cochlear implantation constitutes a successful therapy of inner ear deafness, with the majority of patients showing good outcomes. There is, however, still some unexplained variability in outcomes with a number of cochlear-implant (CI) users, showing major limitations in speech comprehension. The current study used a multimodal diagnostic approach combining single-photon emission computed tomography (SPECT) and electroencephalography (EEG) to examine the mechanisms underlying speech processing in postlingually deafened CI users (*N* = 21). In one session, the participants performed a speech discrimination task, during which a 96-channel EEG was recorded and the perfusions marker ^99m^Tc-HMPAO was injected intravenously. The SPECT scan was acquired 1.5 h after injection to measure the cortical activity during the speech task. The second session included a SPECT scan after injection without stimulation at rest. Analysis of EEG and SPECT data showed N400 and P600 event-related potentials (ERPs) particularly evoked by semantic violations in the sentences, and enhanced perfusion in a temporo-frontal network during task compared to rest, involving the auditory cortex bilaterally and Broca’s area. Moreover, higher performance in testing for word recognition and verbal intelligence strongly correlated to the activation in this network during the speech task. However, comparing CI users with lower and higher speech intelligibility [median split with cutoff + 7.6 dB signal-to-noise ratio (SNR) in the Göttinger sentence test] revealed for CI users with higher performance additional activations of parietal and occipital regions and for those with lower performance stronger activation of superior frontal areas. Furthermore, SPECT activity was tightly coupled with EEG and cognitive abilities, as indicated by correlations between (1) cortical activation and the amplitudes in EEG, N400 (temporal and occipital areas)/P600 (parietal and occipital areas) and (2) between cortical activation in left-sided temporal and bilateral occipital/parietal areas and working memory capacity. These results suggest the recruitment of a temporo-frontal network in CI users during speech processing and a close connection between ERP effects and cortical activation in CI users. The observed differences in speech-evoked cortical activation patterns for CI users with higher and lower speech intelligibility suggest distinct processing strategies during speech rehabilitation with CI.

## Introduction

Cochlear implantation is an established and effective method of treating sensorineural hearing loss (Wilson and Dorman, 2008a, b; Gaylor et al., 2013). Cochlear implants (CIs) bypass the damaged structures of the inner ear by electrical stimulation of the auditory nerve (Wilson and Dorman, 2008a, b). Although cochlear implantation allows open-set speech perception in most of the cases, there is a high variability in CI outcomes (Heydebrand et al., 2007). This variability cannot be completely explained so far (Lazard et al., 2012; Blamey et al., 2013) but seems to be at least partially related to individual differences in the auditory nerve, the position of the implant electrodes, cognitive abilities, and neuronal plasticity (Nadol, 1997; Drennan and Rubinstein, 2008; Lazard et al., 2012; Rönnberg et al., 2013; Sandmann et al., 2015; Finke et al., 2016a). Neuroimaging can help improve the understanding of the individual differences in speech comprehension by providing important insights into the sensory and cognitive processes underlying speech perception in CI users.

Previous studies using electroencephalography (EEG) and event-related potentials (ERPs) in particular have shown differences in cortical speech processing between CI users and normal-hearing (NH) listeners, both at initial sensory and at later higher-level cognitive processing stages (e.g., Hahne et al., 2012; Finke et al., 2016a). In particular, CI users have shown smaller amplitudes of N1 ERPs to speech sounds, indicating smaller assembly or reduced synchronization of activated neurons in the auditory cortex of CI users when compared with NH listeners (Groenen et al., 2001; Aggarwal and Green, 2012). Regarding the later cognitive processing stages, ERPs in response to semantic anomalies (N400) and syntactic violations (P600) have been rarely examined in CI users (Hahne et al., 2012; Henkin et al., 2014; Kallioinen et al., 2016; Vavatzanidis et al., 2018). The N400, reflecting semantic memory use during language comprehension (Kutas and Federmeier, 2000), has been shown to be prolonged in adult CI users when compared with NH listeners (Hahne et al., 2012; Henkin et al., 2014), suggesting a delayed and a more effortful speech processing with the limited CI input (Finke et al., 2016a). However, it is currently unknown whether the N400 can distinguish between CI users who have good versus poor speech recognition, although such a distinctiveness has been previously shown for other auditory ERPs (Soshi et al., 2014; Turgeon et al., 2014).

Positron emission tomography (PET) and single-photon emission computed tomography (SPECT) enable the precise spatial assignment of neuronal activity that underlies speech perception in CI users (Giraud et al., 2001a,b,c; Wong et al., 2002). In general, the spatial resolution of both PET and SPECT enables investigations of neuronal activity (changes) in the auditory cortex and associated brain regions (Abraham and Feng, 2011). Regarding CI users, SPECT has been shown previously to be a suitable tool to objectively evaluate speech comprehension performance (Allen et al., 2004). In particular, different cortical activations were observed for higher and lower CI performers during speech comprehension (Tobey et al., 2004). SPECT has also been suggested to be considered for the presurgical evaluation of prelingually deaf adults being candidates for cochlear implantation (Di Nardo et al., 2013). Furthermore, previous PET studies with CI users have revealed a positive correlation between speech recognition ability and activation in the primary and association auditory cortices (Green et al., 2005). They have also shown a different network recruited for speech processing in proficient and non-proficient CI users, suggesting that activation in both the temporal cortices and the left inferior prefrontal cortex are a prerequisite for successful speech comprehension (Mortensen et al., 2006). On the other hand, in NH listeners, a stronger activation of inferior frontal regions has been related to enhanced listening effort (Davis and Johnsrude, 2003) and to poorer cognitive abilities, in particular lower working memory capacity (Zekveld et al., 2012). Although previous results point to a remarkable influence of cognitive abilities on speech recognition with a CI (Heydebrand et al., 2007), no study so far has examined how individual differences in cognitive abilities and listening effort relate to the different cortical response patterns in proficient and non-proficient CI users.

The combination of SPECT/PET and EEG measurements allows a synergistic examination of speech processing, as it provides the excellent temporal resolution of the EEG and the good spatial resolution of the emission tomography. A few studies so far have used combined sequential SPECT or PET and EEG measurements to study auditory processing in different groups of patients, in particular in patients with mild and moderate Alzheimer’s disease (O’Mahony et al., 1996; Gungor et al., 2005), Schizophrenia (Blackwood et al., 1994; Shajahan et al., 1997; Medved et al., 2001) or obsessive–compulsive disorders (Molina et al., 1995). The results have revealed correlations between ERPs and regional cerebral perfusion, suggesting a connection between disease-related alterations in auditory ERPs and cortical activation (O’Mahony et al., 1996; Gungor et al., 2005).

The principal aim of this study was to contribute to the better understanding of the high variability in CI outcomes. We used, for the first time, a synchronized multimodal SPECT-ERP approach in CI users to thoroughly examine the neuronal activation patterns underlying speech comprehension and their relation to cognitive abilities. The study also aimed to prove the suitability of a typical EEG paradigm for SPECT imaging. CI users with higher and lower speech performance were tested with a semantic-anomaly paradigm to study the N400 ERP in response to sentences with semantic violations (Kutas and Hillyard, 1980a; Lau et al., 2008). It has been previously shown for NH listeners that the left posterior middle temporal gyrus (MTG) is critically involved in N400 generation (for a review, see Lau et al., 2008). Given this finding and previous PET results about speech processing in CI users (Green et al., 2005; Mortensen et al., 2006), we predicted positive correlations between the N400 response and activation in the MTG. We also expected differences in cortical activation in the (pre)frontal, the superior temporal, and the posterior middle temporal regions, i.e., in temporo-frontal networks, between CI users with higher and lower speech recognition ability (Friederici, 2012). In addition, we expect that CI users with higher speech comprehension have stronger activation in regions and networks representing cognitive functions, such as the temporal cortex (memory) and parietal cortex (attention) (Coez et al., 2014). Finally, we tested the hypothesis that cross-modal (i.e., auditory) activation of the occipital (i.e., visual) cortex is beneficial in terms of speech understanding with a CI (Giraud et al., 2001b). Indeed, our results suggest a close connection between ERP effects and cortical activation in CI users and different activation patterns during speech processing between higher and lower performers, pointing to different neural resource allocation and strategies used for speech processing.

## Materials and Methods

In this section, the following methodological issues are described in subchapters: the patient characteristics (*Patients*), the sequence of procedures (*Sequence of Procedures*), the audiometric and cognitive tests (*Audiometric and Neurocognitive Testing*), the applied EEG/SPECT paradigm (*Speech Condition Stimuli for Combined EEG-SPECT Measurement*), and details on EEG (*EEG Recording*) and SPECT (*SPECT – Acquisition and Reconstruction*) acquisition. For the latter two methods, *Data Analysis* gives details on data analysis.

### Patients

Twenty-one postlingually deafened CI users [mean age, 62.1 years; standard deviation (SD), 11.5 years; range, 30–80 years; 10 female) participated in the present study, with 18 CI users being consistent right-handers and three being consistent left-handers (Annett, 1970). Eight CI users were implanted unilaterally (5 left), and 13 were implanted bilaterally. In case of bilateral implantation, the “better” ear, according to the performance in the Freiburg monosyllabic word test (Hahlbrock, 1970), was used for stimulation (5 left). All CI users were native German speakers, had at least 11 months of CI experience (mean, 99.4 months; SD, 68.5; range, 11.0–346.0 months) and achieved a word recognition score of at least 20% in the Hochmair–Schulz–Moser (HSM) sentence test in quiet (Hochmair-Desoyer et al., 1997) with the tested CI. None of the CI users reported using sign language for communication. Details concerning the subject’s implant system and demographics can be obtained in Table 1. None of the CI users reported neurological or psychiatric disorders or used medications affecting the central nervous system.

**TABLE 1:** Patient characteristics.

**Sex**	**Age**	**Handedness**	**Etiology**	**Side of stim.**	**Implant**	**Contralateral to stim. side**	**Age at onset of profound deafness (years)**	**Age at implantation (years)**	**Duration of deafness (months)**	**Implant use (months)**	**Hearing threshold of contralateral ear**
**Lower performers**											
F	30	Left	Unknown	Left	Nucleus CI512	Nucleus CI24RE (CA)	27	27	1	25	≥ 90 dB
F	64	Right	Otitis media	Left	Nucleus CI422	Nucleus CI24RE Hybrid-L (H)	55	59	45	65	≥ 90 dB
F	53	Right	Genetic	Right	AB HiRes90K Helix	AB HiRes90K Helix	31	42	133	124	≥ 90 dB
M	73	Right	Genetic	Left	Nucleus CI512	HA	65	65	1	87	≥ 90 dB
F	52	Right	Unknown	Left	Nucleus CI512	Nucleus CI24R (CA)	23	23	8	346	≥ 90 dB
M	70	Right	Acute hearing loss	Left	Nucleus CI24RE Hybrid-L (no ACO)	deaf	61	61	1	112	≥ 90 dB
M	75	Left	Unknown	Right	Nucleus CI24RE (CA)	Nucleus CI24M	22	62	492	151	≥ 90 dB
M	77	Right	Noise trauma	Right	MED-EL Sonata Flex EAS 20 (Hann.)	MED-EL Sonata Flex EAS 20 (Hann.)	69	69	1	100	80 dB HL 0.25 kHz
F	70	Right	Morbus Menière	Right	Nucleus CI532	HA	65	68	41	13	75–85 dB HL 0.25–2 kHz
M	65	Right	Unknown	Right	Nucleus CI24RE (CA)	Nucleus CI24R (CA)	41	54	157	129	≥ 90 dB
Mean ± SD	62.9 ± 13.6						45.9 ± 18.1	53.0 ± 15.8	88.0 ± 145.2	115.2 ± 87.7	
**Higher performers**											
M	64	Right	Otosclerosis	Right	Nucleus CI24R (CA)	Nucleus CI24RE (CA)	49	51	21	160	≥ 90 dB
F	45	Right	Unknown	Right	Nucleus CI24RE Hybrid-L (no ACO)	Nucleus CI422	39	39	1	79	≥ 90 dB
F	48	Right	Unknown	Left	Nucleus CI24RE (CA)	HA	43	43	1	58	65 dB HL 0.25 kHz
M	65	Right	Morbus Menière	Right	MED-EL Sonata ti100	HA	57	57	1	80	≥ 90 dB
M	59	Left	Unknown	Right	AB HiRes90K Helix	AB Clarion CII	26	49	274	116	≥ 90 dB
M	58	Right	Unknown	Left	MED-EL Concerto Flex EAS 28 (no ACO)	HA	53	53	1	58	45–90 dB HL 0.25–2 kHz
F	68	Right	Acute hearing loss	Left	AB HiRes 90K Advantage HiFokus Mid-Scala	HA	61	63	19	58	40–90 dB HL 0.25–8 kHz
F	60	Right	Unknown	Right	AB HiRes90K Helix	AB HiRes90K Helix	50	51	4	109	≥ 90 dB
F	67	Right	Genetic	Left	AB HiRes90K Helix	Nucleus CI24RE (CA)	56	56	1	129	≥ 90 dB
M	80	Right	Acute hearing loss	Right	Nucleus CI512 Profile (CA)	HA	73	79	66	11	60–80 dB HL 0.25–8 kHz
M	62	Right	Unknown	Left	Nucleus CI522	Nucleus CI422	59	59	1	78	≥ 90 dB
Mean ± SD	61.5 ± 9.1						51.5 ± 11.9	54.5 ± 10.1	35.5 ± 77.7	85.1 ± 39.2	
Overall mean ± SD	62.1 ± 11.5										

**F, female; M, male; HA, hearing aid; stim, stimulation; ACO, acoustic component of the hybrid/electric acoustic stimulation (EAS) device. Age at onset of profound deafness refers to the age at which the amount of hearing loss was too severe to be successfully treated by a conventional hearing aid. Duration of deafness is defined as the time between the age at onset of profound deafness and the CI implantation. Comparing age at onset of profound deafness (years), age at implantation (years), duration of deafness (months), and implant use (months), respectively, between groups of lower and higher performing CI users did not reveal any significant differences between groups (unpaired t-test).**

All participants gave informed written consent before the experiment. The study was approved by the Ethics Committee of the Hannover Medical School (vote number 6678) and the German Federal Office for Radiation Protection (reference number Z5-22461/2-2014-012) and was carried out in accordance with the Declaration of Helsinki.

### Sequence of Procedures

In this subchapter, the sequence of procedures is described. Details on the procedures are given in the following subchapters. The participants underwent two individual sessions, separated by 13.0 ± 6.5 days. In one session, CI users completed the neuropsychological testing, except for the size-comparison span test (SICSPAN) (Sorqvist et al., 2010). Subsequently, a SPECT scan was performed after application of 729.1 ± 8.1 MBq Technetium-99m (^99m^Tc) labeled HMPAO without stimulation (“rest condition”). Injection of the substance took place in a quiet room with dimmed light, where participants stayed for 15 min before and 5 min after application for uptake. The SPECT scan itself was performed ∼1.5 h postinjection (p.i.). In the other session, participants underwent the audiometric testing and the SICSPAN (Sorqvist et al., 2010), which was followed by combined sequential EEG and SPECT measurements (“speech condition”). The participants were seated comfortably in a dimly lit, as well as electrically and acoustically shielded cabin, 100 cm in front of a computer screen. Before the actual start of the EEG experiment, participants performed a training block with seven sentences. Subsequently, the participants listened to 80 different sentences (40 semantically correct/40 semantically incorrect), which were presented within the course of two experimental blocks, with 55 sentences being presented in block 1 (∼7 min) and 25 sentences presented in block 2 (∼3 min). The length of the respective blocks was adapted so that – without interrupting the paradigm – application (2 min after the start of block 1) and subsequent radiopharmaceutical uptake phase (for another 5 min) was possible. The order of the sentences was pseudo-randomized between participants. The CI users were instructed in written form to listen to each sentence while focusing on a black screen. A white fixation point, appearing 1,000 ms after the offset of each sentence, signaled the participants to provide a response via a button press on whether the sentence was semantically correct or not. Assignment of the buttons to the two answer possibilities was counterbalanced across participants. The fixation point remained on the screen for 3,000 ms, which constituted the response window. The delayed response window ensured decoupling the N400 and P600 ERPs from the motor response. After the task, subjects were asked to evaluate the *subjective listening effort* during the task with a 5-point rating scale (1.0 = not demanding, 5.0 = too demanding; the words could not be understood). During the first block, 2 min after the start of the task, 731.5 ± 6.8 MBq ^99m^Tc-labeled HMPAO was applied intravenously via medical tubing from outside the shielded cabin. Approximately 1.5 h after injection, a SPECT scan was acquired, reflecting cortical activity during the sentence discrimination task. In general, the “rest condition” was performed first, and the “speech condition” (combined sequential SPECT and EEG measurements) took place in the second session. Due to organizational issues, however, the “speech condition”’ was carried out first in a few cases (*n* = 4).

### Audiometric and Neurocognitive Testing

Speech recognition abilities obtained with the CI used in the experimental session were assessed using three frequently applied German speech tests: (1) the Freiburg monosyllabic word test in quiet (Hahlbrock, 1970), (2) the HSM sentence test (Hochmair-Desoyer et al., 1997) in quiet and in noise [10 dB signal-to-noise ratio (SNR)], and (3) the Göttinger sentence test (GÖSA; adaptive noise; Kollmeier and Wesselkamp, 1997). The GÖSA is a widely used audiometric test, which contains complete German sentences that reflect the everyday speech situation. It uses an adaptive procedure to measure the signal-to-noise ratio at which 50% of the speech signal is correctly understood. In the current study, the speech material of all of the three speech tests was presented at a sound intensity of 65 dB SPL. Participants were instructed to report all words perceived.

Our study aimed to compare brain activation patterns between CI users with different performance levels. However, the present study did not enable to compare markedly poor and good performers, since all CI users had to at least be able to perform the sentence discrimination task to a certain extent, allowing for an adequate number of correct EEG trials for analysis. Groups, therefore, rather represent CI users with higher or lower performance levels. Accordingly, the group assignment was based on a median split procedure, for which we relied on the GÖSA, resulting in a cutoff of +7.6 dB SNR. This procedure was not based on previous studies, but rather exploratory, with the aim of obtaining groups of CI users with different performance levels. In the following, the groups are referred to as “lower” (50% speech reception threshold at > 7.6 dB SNR; note that more positive values indicate worse performance) and “higher” CI performers (50% speech reception threshold at < 7.6 dB SNR). In the present study, the GÖSA was chosen to be the most appropriate one for the group selection based on the median split procedure, for the following reasons: (1) It contains meaningful sentences from everyday life (ecological validity of stimulus material), (2) it allows the measurement of an individual speech reception threshold by means of an adaptive procedure (speech test with high accuracy), and (3) it is highly demanding and provides performance scores with fair variability in performance scores (no problem of floor and ceiling effects). Thus, with regards to group assignment, the GÖSA test is preferable to the Freiburg monosyllabic word test and the HSM sentence test, given that the GÖSA test uses a more appropriate stimulus material (some of the words in the Freiburg monosyllabic word test are outdated), and the test results are not confounded by floor and ceiling effects (non-adaptive speech tests, for instance the HSM sentence test with a fixed SNR, provide a risk for these boundary effects).

To control for residual hearing, the contralateral device was detached at the time of testing and the ear was closed by means of an earplug. Beforehand, to assess residual hearing in the non-tested contralateral ear, a pure-tone audiometry (unaided; range, 0.25–8 kHz) was performed.

Beside the audiometric tests, participants completed four different cognitive tests, assessing working memory capacity and verbal abilities: (1) The “Mehrfachwahl-Wortschatz-Intelligenz-Test” (MWT-B, Lehrl, 1977) was applied to measure *verbal intelligence*. Here, participants had to identify a real word among four pseudowords. According to the official guidelines provided with the test material, individual percentiles (in relation to a normative sample) were used for the statistical analyses. (2) The lexical verbal fluency subtest of the “Regensburger Wortflüssigkeits-Test” (RWT, Aschenbrenner et al., 2001; Harth et al., 2004) was used to test *verbal fluency*. During this test, subjects were asked to report as many words as possible with the initial letter “s” within 2 min. Here, likewise, individual percentiles in relation to a normative sample provided with the test manual were used for the statistical analyses. (3) A German version of the SICSPAN (Sorqvist et al., 2010; Finke et al., 2016a) was used to assess the verbal *working-memory* capacity. The SICSPAN was analyzed using the total percentage of correctly remembered words. (4) We used two subtests (*verbal learning*, *verbal recall*) of the CERAD-Plus test battery (Memory Clinic Basel)^1^ to assess verbal abilities. *Z*-scores in relation to age-specific normative data were used for statistical analysis.

### Speech Condition Stimuli for Combined EEG-SPECT Measurement

The stimulus material consisted of 87 sentences in German language, constructed out of 6 words each (determinative, subject, the auxiliary “hat/haben”/“has/have,” determinative, object, past participle). The sentences were clearly pronounced by a female speaker, spoken at a moderate pace (213 ± 92 ms between the words). The sentences’ final word was either semantically correct (e.g., “Die Mutter hat den Kuchen gebacken”/“The mother has baked the cake”) or incorrect (e.g., “Der Junge hat das Radio gebadet”/“The boy has bathed the radio”) with regards to the previous sentential context. All critical sentence final words appeared in a correct as well as in an a semantically incorrect sentence, thereby guaranteeing that the integration of the word in the semantic context is responsible for different ERP effects rather than the word itself. All sentences were spoken by a trained female native German speaker. Audio files had a sampling frequency of 44 kHz with a 32-bit resolution. Sentence duration ranged from 4.01 to 5.85 s. The onset of each final word was carefully identified by auditory and visual inspection to ensure an accurate time locking of the N400 and P600 ERPs in response to the final word. Stimuli were delivered using the Presentation software (version 16.5; Neurobehavioral Systems, Inc., Berkeley, CA, United States) running on a personal computer. Sentences were presented via two loudspeakers (HECO victa 301) located at 50° azimuth. In the case of a second CI or a conventional hearing aid at the contralateral side, the device was detached for the duration of the experiment, and the respective ear was closed with a wax earplug. In total, 10 CI users were stimulated on the left and 11 on the right side. Similar to previous studies (e.g., Sandmann et al., 2015; Schierholz et al., 2017), the participants used a 7-point loudness-rating scale, which allowed adjusting the perceived loudness of the sentences to a comfortable level, equivalent to 60–70 dB (Allen et al., 1990; Zeng, 1994).

### EEG Recording

EEG data were recorded using 94 Ag/AgCl electrodes, integrated in an infracerebral electrode cap with an equidistant electrode layout (Easycap, Herrsching, Germany). To record an electrooculogram, two additional electrodes were placed below the two eyes. The reference electrode was positioned on the nose tip. A midline electrode, placed anterior to the frontocentral scalp region (AFz), served as ground. Data were recorded by means of three linked 32-channel BrainAmp amplifiers (BrainProducts, Gilching, Germany), with a sampling rate of 1,000 Hz and an online analog filter from 0.02 to 250 Hz. For data acquisition, electrode impedances were kept below 10 kΩ.

### SPECT – Acquisition and Reconstruction

For the scan, participants were positioned as comfortably as possible on the patient bed, and their head was carefully fixed with a special headband with Velcro straps. The participants were instructed to avoid head movements during the scan. Acquisition was performed using a dual-head SPECT camera (Discovery 670 NM/CT, GE Healthcare, Haifa, Israel) equipped with low-energy high-resolution (LEHR) parallel-hole collimators. In total, 180 projections, that is, 90 projections for each of the two detectors, were acquired using a step and shot mode with circular orbit (rotation around the head of the patient with the smallest possible distance, normally 15 cm). With this setup, typically, a total number of counts in the order of 8–9 million could be achieved per acquisition. The required projection time was individually determined before starting the scan on the basis of the count rate detected with the patients’ head in the camera field of view. Typically, the count rate was between 1.4 and 1.6 kCts, and the according total recording time was about 55 min. Projections were acquired with a 128 × 128 matrix size and a zoom factor of 2.0 (pixel size, 2.23 × 2.23 mm^2^). The quality of unprocessed projection data was assessed visually in cine mode and in the form of sinograms, e.g., with respect to motion artifacts, immediately after the recording. Data were reconstructed iteratively, using an ordered-subset expectation maximization (OSEM) algorithm with 5 iterations, 10 subsets, and a Butterworth filter with a cutoff frequency of 0.55 cycles/cm, power of 10 (Hudson and Larkin, 1994), and a dual window scatter correction (scaling 1.1) (Jaszczak et al., 1984) including attenuation correction according to Chang (threshold for boundary detection of 5% and attenuation coefficient of 0.11/cm) (Chang, 1978).

### Data Analysis

#### EEG Preprocessing

EEG data were preprocessed using custom scripts in MATLAB 9.2.0.556344 (R2017a; Mathworks, Natick, MA) and EEGLAB (version 13.6.5b, Delorme and Makeig, 2004). Raw data were imported, down-sampled to 500 Hz, and low-pass filtered (40 Hz) using a Hann-windowed zero-phase finite impulse response (FIR) filter implemented in EEGLAB (pop firws.m; Widmann and Schröger, 2012). Electrodes covering the CI speech processor as well as the transmitter coil were omitted for recording and accordingly removed for the analysis. Subsequently, the continuous data were segmented into 2-s segments and pruned for unique, non-stereotype artifacts. The remaining data were high-pass filtered (1 Hz; Hann-windowed FIR filter; pop_firws.m) and subjected to an extended infomax-independent component analysis (ICA, Bell and Sejnowski, 1995). The resulting ICA weights were applied to the raw data that were filtered (0.1–30 Hz; Hann-windowed FIR filter; pop_firws.m) and epoched (-250–7,950 ms) relative to sentence onset. The prestimulus interval (-250–0 ms) was used for baseline correction. ICA components representing eye blinks, horizontal eye movements, heartbeat activity, and CI artifacts were identified and removed (mean, 36.7%; *SD* = 14.5%; Jung et al., 2000a, b; Debener et al., 2008). Regarding the latter, we identified the components representing the CI artifact by the centroid on the side of the implanted device and by the pedestal artifact in the time course of the respective component (Sandmann et al., 2009, 2010, 2015). Missing channels were interpolated using a spherical spline (mean, 5.8; SD, 1.6; range, 2–10 electrodes). Additional triggers were set, marking the onset of the final word (correct, semantic violation) in each sentence. Based on these triggers, additional epochs, time locked to the onset of the final words (−250–950 ms) were created. Data were corrected using the time interval of −250–0 ms relative to the onset of the critical word.

#### EEG Data Analysis

**FIGURE 1:**
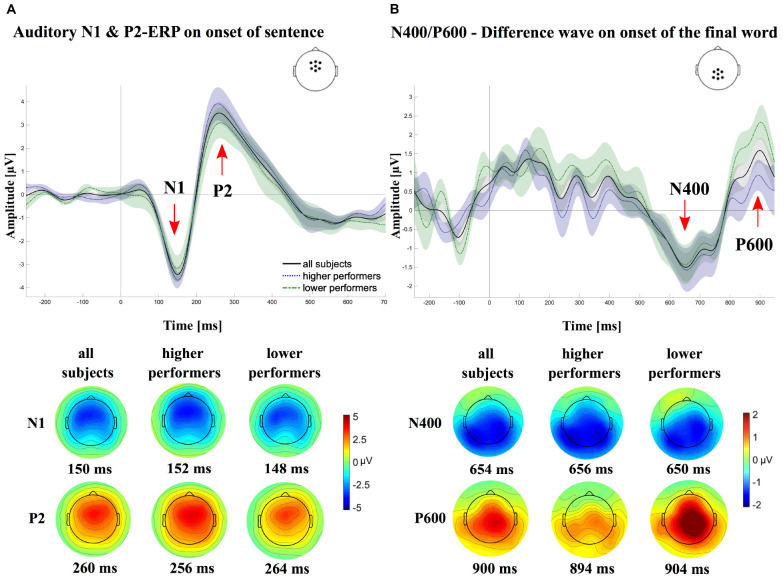
Grand averages of the event-related potentials (ERPs), once for all subjects (*N* = 21; solid lines) and once separately for the groups of higher (*N* = 11; dotted lines) and lower (*N* = 10; dashed lines) cochlear-implant (CI) performers. **(A)** Shows the grand average ERPs in response to the sentence onset for a centrofrontal electrode region of interest (ROI). A clear negative (∼150 ms) and a clear positive (∼240 ms) deflection, referred to as the N1 and P2, respectively, can be observed for all three ERPs. **(B)** Shows the average difference waves (ERPcritViol - ERPcritCorr), time locked to the onset of the critical words, using a centroparietal electrode ROI. A negative (N400) and positive deflection (P600) can be observed for all three ERP waves around 650 ms. At the bottom of each panel, the topographical voltage maps are displayed for the time of the respective peak latencies of the different components [N1, P2 **(A)**, N400 and P600 **(B)**, separately for the different average ERPs (all, higher performers, lower performers).

Single-subject ERPs were computed to the *onset of the sentences* by averaging over all correctly categorized trials, irrespective of the condition (correct, semantic violation) of the sentence (ERP_onset_). Additionally, ERPs to the *onset of the final word* were computed for each participant, separately for semantically correct sentences (ERP_critCorr_) and sentences with a semantic violation (ERP_critViol_), including only the correctly identified trials (critCon: mean, 87.3%; SD, 6.6%; critViol: mean, 87.5%; SD, 7.3%). Furthermore, a difference wave was computed relative to the onset of the final word (ERP_critDiff_ = ERP_critViol_ - ERP_critCorr_). The single-subject ERPs to sentence onset were analyzed using a fronto-central region-of-interest (ROI), including seven electrodes around FCz (see Figure 1A), and a time window of the auditory N1 and P2 (N1, 80–200 ms; P2, 160–280 ms), determined by visual inspection of the grand average ERP and based on previous studies with CI users (see, e.g., Finke et al., 2015, 2016b). Regarding the final word onset, the N400 and the P600 ERPs were analyzed by considering a centroparietal ROI around CPz for both the N400 (time window, 300–900 ms; seven electrodes) and the P600 (time window, 750–940 ms; seven electrodes, see Figure 1B). Both time windows were defined by visual inspection of the grand average ERPs and based on previous studies with CI users (Hahne et al., 2012). For the quantification of the evoked responses, we determined the local minimum (N1, N400) or the local maximum (P2, P600), respectively, of the ERP amplitudes in the respective time window and the respective ROI (peakdet.m)^2^. The mean amplitude was computed for ± 10 ms around the local minimum/maximum. The latency of the respective peaks was determined by detecting the time of the local peak minimum (N1, N400) or maximum (P2, P600). Amplitude and latency measures were subjected to correlation analyses with the SPECT data. Moreover, ERPs were compared between a group of higher and a group of lower performing CI users by means of independent *t*-tests, separately for each ERP components.

#### SPECT Data Analysis

SPECT images were analyzed using the statistical parametric mapping software (SPM8, Wellcome Trust Center for Neuroimaging, Institute of Neurology, University College London, London, United Kingdom), running within MATLAB 9.2.0.556344 (2017a; Mathworks, Natick, MA, United States). First baseline (“rest condition”) and stimulation (“speech condition”) images of each patient were realigned and transformed into a standard stereotaxic anatomical space according to the Montreal Neurological Institute (MNI) employing the default brain perfusion SPECT template provided in SPM8. Further preprocessing included scaling of the images before statistical testing. In order to compare the speech and the rest condition, images were scaled to the 75th percentile (Buchert et al., 2006). Then, effects of stimulus presentation were assessed using a paired *t-*test. For further group comparisons and correlations to speech audiometry and EEG, difference images were generated based on procedures included in subtraction ictal SPECT coregistered to MRI (SISCOM) analysis (Huberfeld et al., 2006; Apostolova et al., 2008), in particular a two-step scaling procedure. First, images were scaled to the global average, using a gray matter mask excluding the cerebellar voxels. Then, preliminary difference images (speech condition minus rest condition) were created. Proceeding from these, a mean value of voxels with a low difference between speech condition and rest condition (i.e., <2 times the standard deviation of the mean) was calculated. This mean value was used for rescaling the speech condition study, which avoids an impact of voxels from activated areas on scaling. Thereafter, final difference images were calculated by subtracting rest condition images scaled to global average from rescaled speech condition images. These final *difference images* (speech condition minus rest condition) were used for further group comparisons and correlations. Moreover, for the assessment of group difference and correlations based on *baseline images* (rest condition), these images were scaled to the 75th percentile.

For image-based statistics, SPM8 was used as well. First of all, results of statistical tests presented here were generated using a significance level of *p* < 0.001 (uncorrected for multiple comparisons) for inferences. The threshold was chosen with respect to previous studies, in particular studies of central auditory processing via auditory implants, where this threshold has been successfully employed (Giraud et al., 2001c; Coez et al., 2009; Berding et al., 2015; Mamach et al., 2018). Furthermore, it has been proposed as a good compromise compensating for the limited sensitivity of brain perfusion SPECT, whereby it is still protecting from false positive results (Signorini et al., 1999). Results are listed without extent voxel threshold (*k* = 0) in the tables. For displaying results, however, two different voxel thresholds were employed. This was done in order to account for different magnitudes in cluster sizes observed across test results.

For test results with a relatively small size of the largest cluster, an extent voxel threshold of *k* = 19 was used. Corresponding results are displayed using the so-called glass brain visualization. For all other test results including relatively large cluster sizes, an operational extent voxel threshold of *k* = 50 was used. Corresponding results are presented using surface rendered MRI image in MNI space. In general, the “modern design” option provided by SPM was employed to include some information on the depth of localized activations. Moreover, locations of significant differences were spatially assigned by automated anatomical labeling, specifically by overlaying the statistical parametric map with a Brodmann volume of interest (VOI) atlas (Rorden and Brett, 2000; Tzourio-Mazoyer et al., 2002). Additionally, statistical analyses were performed including correction for multiple comparisons based on the family-wise error (FWE) rate procedure, together with a cutoff of *p* < 0.05 and an extent threshold of *k* = 0 for statistical inferences (Flandin and Friston, 2019). However, this is a quit conservative approach for the correction for multiple comparison, and nevertheless, analyses without correction for multiple comparison as described above can be regarded as justified particularly in the context of preexisting according *a priori* hypotheses.

To assess potential effects of handedness, paired *t*-tests comparing speech and rest condition were performed twice, once including and once excluding the three left-handed participants. As no differences were obvious in the resulting statistical parametric maps, all left-handers were included in the further analyses. Another potentially influencing factor is the side of stimulation (right = 11; left = 10). Therefore, an additional comparison of speech and rest condition was performed, with images from patients with left-ear stimulation being flipped in the mid-sagittal plane. Only minor differences for flipped vs. non-flipped images were observed in primary and secondary auditory cortices. Therefore, the original, non-flipped images were used for all other analyses in order to avoid confusion with primarily unilateral components of brain networks related to speech processing (like, e.g., Broca’s area).

Further analyses included the comparison of the cortical baseline activity (baseline images) and stimulated activation (difference images) between CI users with higher and lower performance in speech comprehension. Similar to the EEG data analysis, the subgroups of higher and lower CI performance were compared by means of two-sample *t*-tests in consideration of two different contrasts [(1) lower performer > higher performers and (2) higher performers > lower performers]. Additionally, correlation analyses were performed using SPM to explore for the relationship between difference images on the one hand and EEG, audiometric, as well as neuropsychological data on the other hand.

## Results

### Speech Comprehension Ability and Cognitive Functions

Results for speech audiometry, cognitive tests, and speech task performance are listed in Table 2 with separate means for patients with higher and lower speech comprehension as well as the results from group comparisons across the different tests. In the context of speech audiometry, the CI users showed overall high performance for speech recognition in quiet, with average scores of 81.7 ± 12.1% (Freiburg monosyllabic word test) and 93.0 ± 11.0% (HSM sentence test without background noise). As expected, speech recognition in noise was remarkably lower, as revealed by the average recognition score of 48.5 ± 19.1% in the HSM sentence test (10 dB SNR) and the average 50% speech reception threshold of + 8.6 ± 4.2 dB SNR in the GÖSA.

**TABLE 2:** Results of speech audiometry, cognitive tests, and sentence-discrimination task during EEG recording.

	**Speech audiometry**				**Cognitive testing**					**Sentence-discrimination taskance**		
	**Freiburg monosyllabic word test (%)**	**HSM sentence test in quiet (%)**	**HSM sentence test in noise (10 dB SNR) (%)**	**Göttinger sentence test (dB SNR for SRT50%)**	**Verbal intelligence (percentile rank)**	**Verbal fluency (percentile rank)**	**Working memory (% total score)**	**Verbal learning (*Z*-score)**	**Verbal recall (*Z*-score)**	**Hits (%)**	**Correct rejections (%)**	**Subjective listening effort**
	**Lower performers**											
	90.0	95.0	33.0	7.9	32.5	1.0	54.8	−0.3	0.3	88.0	98.0	2.0
	90.0	98.0	29.0	17.6	88.6	31.0	56.5	−1.2	−0.6	90.0	83.0	2.0
	65.0	78.0	11.4	8.8	48.9	70.0	59.7	0.1	−0.1	90.0	98.0	1.0
	80.0	98.0	50.0	11.8	94.3	86.0	56.5	0.8	1.7	95.0	100.0	2.0
	95.0	98.0	55.0	15.4	43.5	60.0	53.2	−0.1	0.1	83.0	88.0	1.0
	65.0	53.8	11.3	15.5	35.8	48.0	27.4	−2.3	−1.6	78.0	78.0	2.0
	75.0	77.0	18.7	14.2	94.3	58.0	66.1	−1.4	−0.1	80.0	98.0	2.0
	75.0	97.0	56.0	10.8	94.3	97.0	37.1	1.2	1.4	90.0	88.0	2.0
	90.0	99.0	81.0	9.8	97.7	58.0	53.2	−0.7	−1.5	95.0	95.0	1.0
	85.0	100.0	75.0	10.6	80.6	50.0	46.8	0.2	−0.5	98.0	98.0	3.0
Mean ± SD	81.0 ± 10.2	89.4 ± 14.4	42.0 ± 23.9	12.2 ± 3.1	71.1 ± 25.9	55.9 ± 25.6	51.1 ± 10.8	−0.4 ± 1.0	−0.1 ± 1.0	88.7 ± 6.3	92.4 ± 7.2	1.8 ± 0.60
	**Higher performers**											
	100.0	100.0	37.0	4.3	97.7	31.0	33.9	−1.8	−1.2	98.0	100.0	2.0
	95.0	100.0	44.0	7.6	48.9	13.0	53.2	−0.6	1.0	98.0	95.0	1.0
	95.0	98.0	49.0	5.7	94.3	79.0	61.3	−1.1	−0.3	100.0	100.0	2.0
	100.0	100.0	68.0	4.5	80.6	87.0	62.9	1.0	−0.6	98.0	83.0	2.0
	75.0	100.0	56.0	2.7	32.5	25.0	37.1	0.6	−0.3	100.0	98.0	1.0
	75.0	97.0	74.0	4.6	71.1	76.0	66.1	1.3	1.6	95.0	98.0	1.0
	70.0	99.0	61.0	7.1	94.3	86.0	48.4	−0.8	−0.1	98.0	95.0	2.0
	60.0	92.0	46.0	5.5	62.2	7.0	38.7	−2.6	−1.8	98.0	98.0	1.0
	85.0	98.0	60.0	2.9	55.0	58.0	61.3	0.4	1.5	98.0	100.0	1.0
	65.0	85.0	48.0	7.2	62.2	98.0	33.9	1.1	1.3	95.0	93.0	1.0
	85.0	91.0	56.0	6.2	29.2	31.0	46.8	1.3	0.4	80.0	85.0	2.0
Mean ± SD	82.3 ± 13.6	96.3 ± 4.7	54.5 ± 10.5	5.3 ± 1.6	66.2 ± 22.9	53.7 ± 31.6	49.4 ± 11.7	−0.1 ± 1.3	0.1 ± 1.1	96.2 ± 5.3	95.0 ± 5.6	1.5 ± 0.5
Sign.	0.82	0.16	0.15	< 0.0001	0.67	0.87	0.74	0.63	0.64	0.01	0.39	0.19

**Significant differences between groups of lower and higher performers have been detected for scores for the Göttinger sentence test (p < 0.0001, unpaired t-test) and the hit rates (%) in the sentence-discrimination task during EEG recording (p = 0.01). dB, decibel; SNR, signal to noise; SRT, speech reception threshold, subjective listening effort with a 5-point rating scale (1 = not demanding, 5 = too demanding; the words could not be understood).**

Performance scores in the cognitive tests showed for the MWT-B (verbal intelligence) an average percentile rank of 68.5 ± 24.5; for the SICSPAN (working memory capacity), an average of 50.2 ± 11.3 (total sum); and for the RWT (verbal fluency), an average percentile rank of 54.8 ± 28.9. *Z*-scores for verbal learning were on average of −0.2 ± 1.2 and for verbal recall of 0.0 ± 1.0.

The performance in the semantic-anomaly paradigm was generally high, with mean hit rates of 92.6 ± 6.9% (correct identification of semantically correct sentences) and mean correct rejection rates of 93.8 ± 6.6% (correct identification of sentences with semantic violations). Comparing the performance in the semantic-anomaly paradigm between CI users with lower and higher speech reception thresholds (median split with cutoff + 7.6 dB SNR in the Göttinger sentence test) revealed reduced hit rates in speech comprehension ability for the lower as compared to the higher CI performers [88.7 ± 6.3% vs. 96.2 ± 5.3%; *t*(19) = 2.8, *p* < 0.05, *r* = 0.5]. The number of *correct rejections* in contrast was not different between groups [92.4 ± 7.2% vs. 95.0 ± 5.6%; *t*(19) = -0.9, *p* = 0.39, *r* = 0.2].

The subjective rating of the listening effort during the EEG task was relatively low, as indicated by the ratings of 1.6 ± 0.6 on average. Comparison of scores for cognitive tests (tested in an unpaired *t*-test) and listening effort between groups of lower and higher performers (using the Mann–Whitney *U*-test) did not reveal any statistical significant differences [SICSPAN: *t*(19) = 0.3, *p* = 0.74, *r* = 0.1; MWT-B: *t*(19) = 0.4, *p* = 0.67, *r* = 0.1; RWT: *t*(19) = 0.2, *p* = 0.87, *r* = 0.1; CERAD verbal learning: *t*(19) = -0.5, *p* = 0.64, *r* = 0.1; CERAD verbal recall: *t*(19) = -0.5, *p* = 0.64, *r* = 0.1 and listening effort, *U* = 39, *p* = 0.28, *r* = 0.24].

### EEG Components

Figure 1 shows the group average ERPs separately for the onset of the sentence (Figure 1A) and the onset of the final word of the sentence (Figure 1B).

A clear negative peak can be observed around 150 ms (N1 peak), followed by a positive deflection around 240 ms (P2 peak). On a group level, the N1 showed a mean amplitude of -4.6 ± 1.6 μV with a mean peak latency of 154.1 ± 14.7 ms. Average values for the P2 mean amplitude and peak latency were 4.1 ± 2.0 μV and of 244.7 ± 19.3 ms, respectively. Independent *t*-tests revealed no significant differences between groups with lower and higher CI performance for N1/P2 amplitudes [N1: *t*(19) = 0.8, *p* = 0.45, *r* = 0.2; P2: *t*(19) = -1.1, *p* = 0.27, *r* = 0.3] and latencies [N1: *t*(19) = 0.8, *p* = 0.45, *r* = 0.2; P2: *t*(19) = 0.8, *p* = 0.45, *r* = 0.2]. Sequential two-tailed *t*-tests, using a sliding window of 2 ms at α = 0.5% were used to compare ERPs on sentence onset between groups of lower and higher CI performers, whereby an interval was considered as significantly different between groups if at least 10 consecutive data points reached a *p* < 0.05 (Guthrie and Buchwald, 1991). To control for multiple comparisons, *p*-values were adjusted using the false discovery rate (FDR; Benjamini and Hochberg, 1995). Results showed no statistical difference for time windows of the N1 and the P2, although on the descriptive level, the N1 and P2 peaks of lower performers were reduced compared with the higher performers (lower vs. higher performer; N1: 80–200 ms, *p* ≥ 0.99, corrected; P2: 160–280 ms, *p* ≥ 0.99, corrected).

Difference waves on the onset of the final word (ERP_critViol_ - ERP_critCorr_) are shown in Figure 1B, using a centroparietal ROI, respectively. A slow negative deflection can be observed between 500 and 800 ms, referred to as the N400. The average mean amplitude on a group level was -3.6 ± 1.6 μV with mean peak latencies of 593.5 ± 132.7 ms. Around 900 ms, a positive deflection (P600) with an average mean amplitude of 3.0 ± 1.5 μV and a mean peak latency of 867.8 ± 41.7 ms can be detected. Independent *t*-tests revealed no significant differences between groups with lower and higher CI performance for N400 amplitudes [*t*(19) = 0.9, *p* = 0.36, *r* = 0.2] and latencies [*t*(19) = 0.2, *p* = 0.85, *r* = 0.1]. For the P600, results, however, revealed a significant difference for P600 amplitudes [*t*(19) = 2.1, *p* = 0.05, *r* = 0.4], but not for latencies [*t*(19) = -0.1, *p* = 0.89, *r* = 0.02]. Sequential two-tailed *t*-tests, using a sliding window of 2 ms at α = 0.5% were used to compare ERPs on the onset of the critical word between groups of lower and higher CI performers, whereby an interval was considered as significantly different between groups if at least 10 consecutive data points reached a *p* < 0.05 (Guthrie and Buchwald, 1991). To control for multiple comparisons, *p*-values were adjusted using the FDR (Benjamini and Hochberg, 1995). Results showed no statistical difference for the time window of the N400, although on the descriptive level, the N400 of lower performers was reduced compared with the higher performers (300–900 ms, *p* ≥ 0.64, corrected). For the time window of the P600, lower performers compared to higher ones, descriptively, revealed elevated P600 peaks, which however could not be verified by sequential two-tailed *t*-tests, when correcting for multiple comparisons (750–940 ms, *p* ≥ 0.64, corrected).

### Comparison of Brain Perfusion: Rest Condition vs. Speech Condition

**FIGURE 2:**
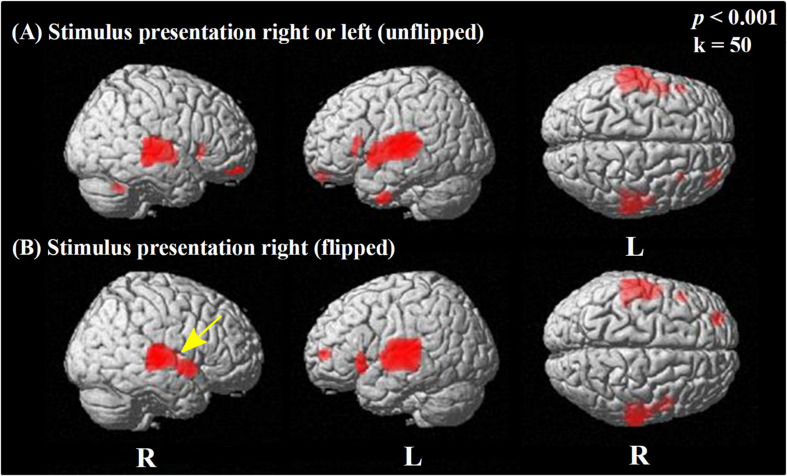
Statistical parametric maps (SPMs) reflecting relative increases of perfusion due to performing the speech condition in comparison to the rest condition. SPMs are overlaid to a surface rendered MRI data set in the Montreal Neurological Institute (MNI) space. There are some areas displayed more transparent than others, which refers to their distance to the projection surface. **(A)** The results obtained using the original data without left/right flipping are displayed. **(B)** To explore for potential effects of the side of stimulation, each data set with left-sided stimulation was flipped in the median sagittal plane, while data sets with right-sided presentations remained unflipped. This resulted uniformly in data sets with stimulation from the “right” side in relation to the images for analysis. For both comparisons, the contrast speech condition > rest condition is displayed. Strong significant perfusion increases due to the task are similarly visible in **(A,B)**, showing bilateral activation in the superior and middle temporal cortices and the inferior prefrontal cortex. Note the minor difference between unflipped and flipped images, with a lack of perfusion increase in the ipsilateral (right) primary auditory cortex (BA 41; yellow arrow) for the flipped in contrast to the unflipped image (see also Supplementary Table A.2).

The activation pattern induced by the sentence discrimination task (speech condition > rest condition) is displayed in Figure 2 (employing a threshold of *p* < 0.001 uncorrected for multiple comparisons). In Figure 2A, the results of a voxel-wise paired *t*-test are shown using the original, non-flipped data, whereas Figure 2B shows the results of the paired *t*-test including flipped images (left to right reversed) for patients stimulated on the left side.

Regarding the *original, non-flipped data* (Figure 2A), the paired *t*-test between the speech and rest condition revealed a strong bilateral activation in the temporal lobe, including the superior [STG; Brodmann areas (BAs) 22, 41, 42], the middle (MTG; BA 21), and the inferior temporal gyrus (ITG; BA 20), as well as the temporo-polar (BA 38) area.

Furthermore, the paired *t*-test showed significant activations in Broca’s area (BA 45 left), bilaterally in the pars orbitalis (BA 47), and the orbitofrontal cortex (BA 11) (Figure 2A) as well as in smaller areas of the left premotor cortex (BA 6) (Supplementary Table A.1.1).

Regarding the paired *t*-test for the *flipped data*, we observed significant activations in the bilateral STG (BA 22, 42), left BA 41, bilateral temporo-polar (BA 38), and left frontal areas (BAs 10, 11, 46, 47), as well as in left BA 45 (Figure 2B), which is bilaterally activated without using an extent voxel threshold (*p* < 0.001, *k* = 0, Supplementary Table A.2.1). Here, also small area activations for the left motor cortex (BA 6) have been detected. However, no activation could be detected at that level of significance in the right primary auditory cortex (BA 41) with stimulation always from the (ipsilateral) right side. The analyses of either data (non-flipped and flipped) including correction for multiple comparisons (FWE) revealed significant activations induced by stimulation only in temporal regions at a level of *p* < 0.05, specifically, on the right (BAs 22, 21) and left side (BA 42) for non-flipped data and the left (BAs 42, 48, 22) and the right side (BAs 22, 21) for the flipped data (Supplementary Tables A.1.2, A.2.2).

### Contrasting Groups of Higher and Lower CI Performance in Speech Comprehension

#### Difference Image

**FIGURE 3:**
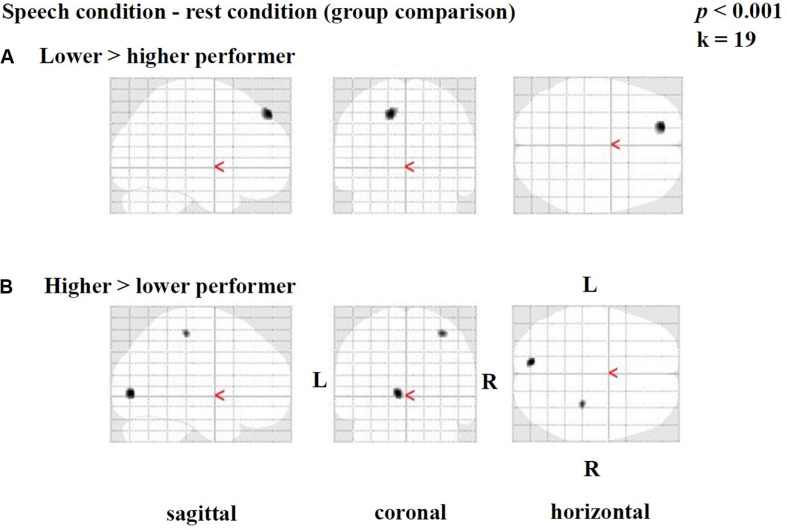
Statistical parametric maps (SPMs) reflecting group differences between higher and lower cochlear-implant (CI) performers with regards to speech-related activation in the context of a semantic-anomaly paradigm. Relative perfusion increases (activations) due to performing the sentence discrimination task are shown. Note a pattern of a **(A)** prefrontal perfusion increase in lower compared to higher performers and **(B)** an increased occipital and parietal perfusion in higher compared to lower performers.

**FIGURE 4:**
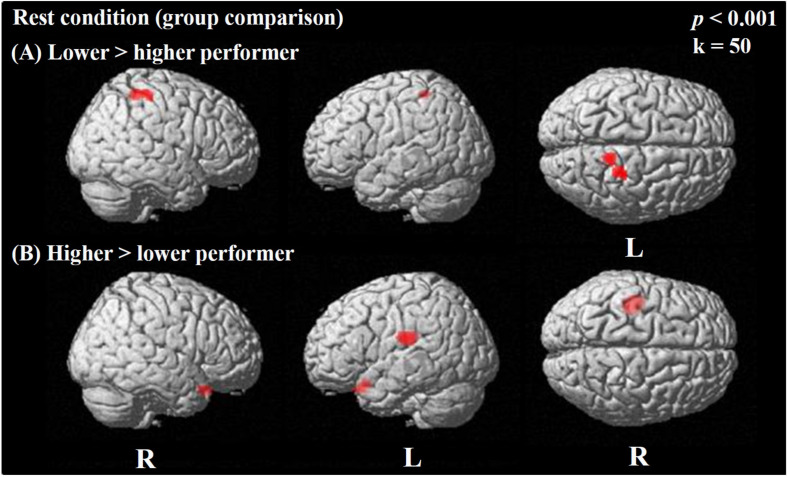
Statistical parametric maps (SPMs) reflecting relative differences in baseline perfusion between groups of cochlear-implant (CI) users with higher and lower performance in speech comprehension according to the GÖSA test (median-split procedure). SPMs are overlaid to a surface rendered MRI data set in the Montreal Neurological Institute (MNI) space. There are some areas displayed more transparent than others, which refers to their distance to the projection surface. Note a pattern of higher baseline perfusion in right parietal areas and motor cortex in CI users with lower performance compared to higher performers **(A)**, while a pattern of higher baseline perfusion in hippocampal and inferior frontal areas is seen in higher as compared to lower CI performers **(B)**.

Group comparisons were performed based on the difference images (speech condition - rest condition) (Table 3 and Figure 3) and the rest condition images (Table 4 and Figure 4). The following results were obtained employing a threshold of *p* < 0.001 uncorrected for multiple comparisons. Group comparisons including correction for multiple comparisons described in *Contrasting Groups of Higher and Lower CI Performance in Speech Comprehension* did not reveal any suprathreshold voxel with *p* < 0.05. During the *speech comprehension task*, lower compared to higher performers showed a significantly higher activation in the left frontal BA 9 (Figure 3A). Smaller areas of activation were seen in the left ITG (BA 20), as well as in the right frontal BA 8 (Table 3). In contrast, higher compared to lower performers showed significantly higher activation in the left occipital area (BA17), as well as in the right parietal (BA 3) and temporal (BA 20) areas (Figure 3B and Table 3).

**TABLE 3:** Results of unpaired *t*-tests comparing the difference images (speech condition - rest condition) between CI users with lower and higher speech comprehension (significance level used for inferences at a voxel level *p* < 0.001, extent voxel threshold *k* = 0).

**Brain region**	**Corresponding Brodmann area**	**Hemisphere**	**x^a^**	**y^a^**	**z^a^**	**T**	**Nb voxel cluster**	**% Cluster**
**Lower CI performer > higher CI performer**								
Frontal	BA 9	L	−16	50	48	4.6	71	19.7
Frontal	BA 8	R	24	18	46	4.1	14	35.7
Inferior temporal	BA 20	L	−44	−2	−48	3.7	1	100.0
**Higher CI performer > lower CI performer**								
Occipital	BA 17	L	−10	−82	0	4.1	44	2.3
Parietal	BA 3	R	32	−30	56	4.0	20	5.0
Inferior temporal	BA 20	L	−52	−34	−30	3.8	2	50.0

**^*a*^MNI coordinates.**

**TABLE 4:** Results of unpaired *t*-test comparing the rest condition image between cochlear-implant (CI) users with lower and higher speech comprehension (significance level used for inferences at a voxel level *p* < 0.001, extent voxel threshold *k* = 0).

**Brain region**	**Corresponding Brodmann area**	**Hemisphere**	**x^a^**	**y^a^**	**z^a^**	**T**	**Nb voxel cluster**	**% Cluster**
**Lower CI performer > higher CI performer**								
Parietal	BA 2	R	12	−42	58	4.3	85	69.4
Parietal	BA 5	R	12	−42	58	4.3	85	3.5
Motor cortex	BA 4	R	26	−30	56	4.0	77	90.9
Motor cortex	BA 6	R	26	−30	56	4.0	77	7.8
Parietal	BA 3	R	26	−30	56	4.0	77	1.3
Motor cortex	BA 6	R	2	−22	58	3.9	42	81.0
Motor cortex	BA 4	R	2	−22	58	3.9	42	4.8
**Higher CI performer > lower CI performer**								
Hippocampal	BA 48	L	−40	−24	22	5.3	242	2.1
Frontal	BA 25	L	−12	16	−22	4.9	231	60.2
Inferior frontal	BA 11	L	−12	16	−22	4.9	231	26.8
Inferior temporal	BA 20	L	−26	−8	−10	4.2	43	74.4
Temporal	BA 34	L	−26	−8	−10	4.2	43	7.0
Frontal	BA 47	R	36	26	−18	4.1	42	90.5
Temporopolar	BA 38	R	36	26	−18	4.1	42	2.4
Frontal	BA 47	L	−32	30	−18	3.9	28	28.6
Inferior frontal	BA 11	L	−32	30	−18	3. 9	28	3.6

**^*a*^MNI coordinates.**

#### Rest Condition Image

During the *rest condition*, CI users with lower performance demonstrated significantly higher activity in the right motor and premotor cortex (BAs 4, 6) as well as the right parietal regions (BAs 2, 3, 5) (Figure 4A and Table 4). However, the group of CI users with higher performance showed significantly higher baseline activity in the left hippocampal area (BA 48) and left inferior frontal areas (BA 11, 25) as shown in Figure 4B. Additionally, a higher resting-state perfusion in higher compared to the lower performers was detected in smaller areas of right temporo-polar area (BA 38), the frontal cortex BA 47 (bilateral), and the left inferior temporal cortex (BA 20) (Table 4).

### Correlation Analyses

#### Brain Activation in SPECT vs. Audiometric and Cognitive Performance

**FIGURE 5:**
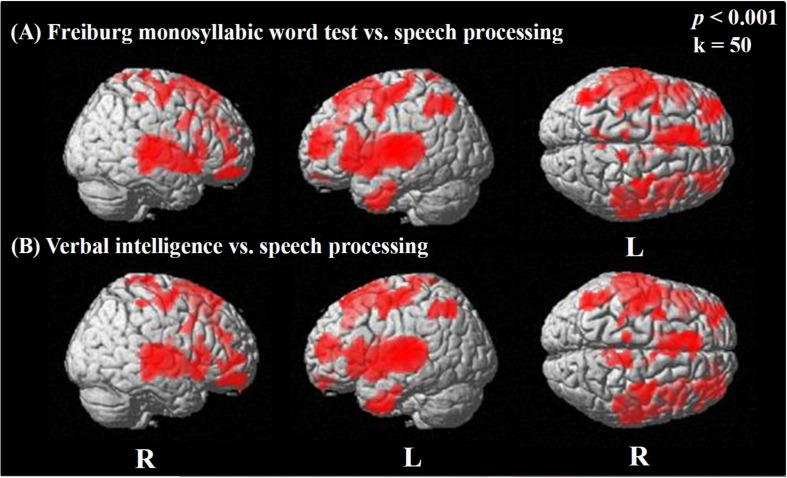
Statistical parametric maps (SPMs) reflecting correlations between activation during the speech-discrimination task (semantic-anomaly paradigm) and the result of the Freiburg monosyllabic word test (speech recognition; **A**) and the MWT-B (verbal intelligence; **B**), respectively. SPMs are overlaid to a surface rendered MRI data set in the Montreal Neurological Institute (MNI) space. There are some areas displaying more transparency than others, which refers to their distance to the projection surface. Note the extended activation of bilateral areas in temporal, frontal, and parietal cortices, showing significant relationships with speech processing (during task) and verbal intelligence.

We observed widespread positive correlations between activation in the difference image (speech condition minus rest condition) and the results of the Freiburg monosyllabic word test (Figure 5A; see also Supplementary Table A.3.1) and the MWT-B, assessing the verbal intelligence (Figure 5B; see also Supplementary Table A.4.1). Both correlations showed a similar distribution pattern of significantly activated regions, including broad bilateral temporal (BAs 20, 21, 22, 38, 41, 42), frontal (BAs 9, 10, 11, 44, 45, 46, 47), and parietal areas (BA 1, 2, 3, 40), as well as the bilateral motor cortex (BAs 4, 6) (Figures 5A,B). These results for the Freiburg monosyllable test and the MWT-B test were obtained employing a threshold of *p* < 0.001 uncorrected for multiple comparisons. Including correction for multiple comparisons (FWE) and using a threshold of *p* < 0.05 restrained the observed significances to the temporal cortices. Specifically, significant correlations were detected with the Freiburg test on the right (BA 22, 21) and left side (BA 42, 48, 22) and the MWT-B on the left (BA 42, 48, 22, 21, 20) and the right side (BA 48, 22, 21) (Supplementary Tables A.3.2, A.4.2).

**FIGURE 6:**
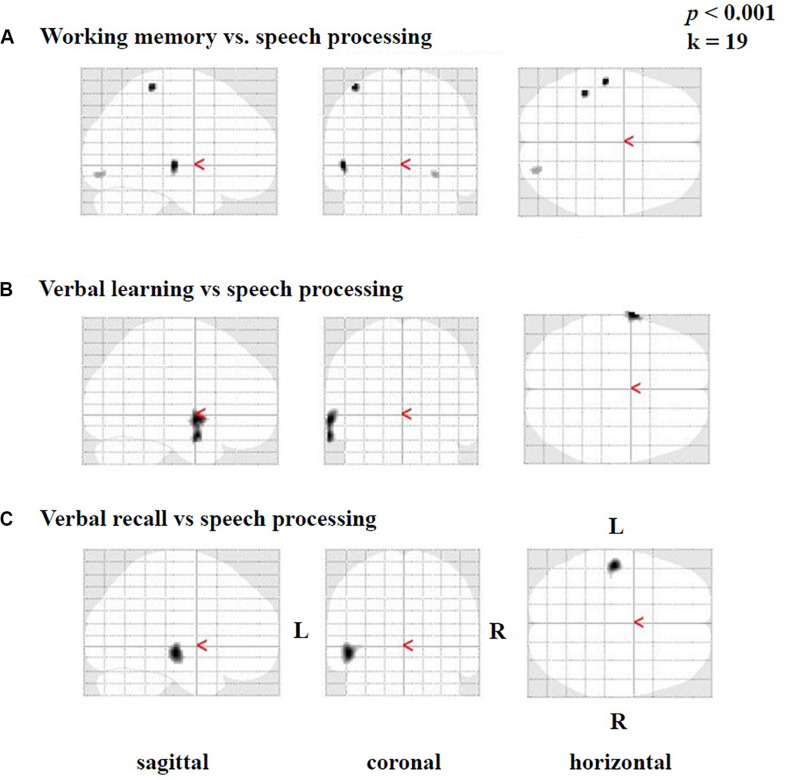
Statistical parametric maps (SPMs) reflecting correlations between activation during the speech-discrimination task (semantic-anomaly paradigm) and cognitive tests, in particular **(A)** working-memory capacity, **(B)** verbal learning and **(C)** verbal recall. Note vastly predominant left temporal correlations for all three tests.

Furthermore, we found smaller areas of positive correlations [only in testing without correction for multiple comparisons and not in tests including correction (FWE)] between the difference image (speech condition minus rest condition) and the results of the SICSPAN test for working memory (Figure 6A), as well as with verbal learning (Figure 6B) and verbal recall (Figure 6C). Specifically, higher capacity in working memory (SICSPAN test) correlated with enhanced perfusion in the left STG (BA 22), MTG (21), and ITG (BA 20), as well as in left parietal (BAs 2, 3) and right occipital regions (BAs 18, 19) (Figure 6A, see also Supplementary Table A.5). With regards to verbal learning, higher *Z*-scores were related to higher perfusion in the left STG (BA 22), MTG (BA 21), and the temporopolar area (BA 38) (Figure 5B; see also Supplementary Table A.6). Finally, a better performance in the verbal recall was associated with a higher activation in the left STG (BA 22) MTG (BA 21), and ITG (BA 20) (Figure 6C, see also Supplementary Table A.7). In the Supplementary Tables A.5–7, the results are listed without application of an extent voxel threshold (*k* = 0).

#### Brain Activation in SPECT vs. EEG Components

**FIGURE 7:**
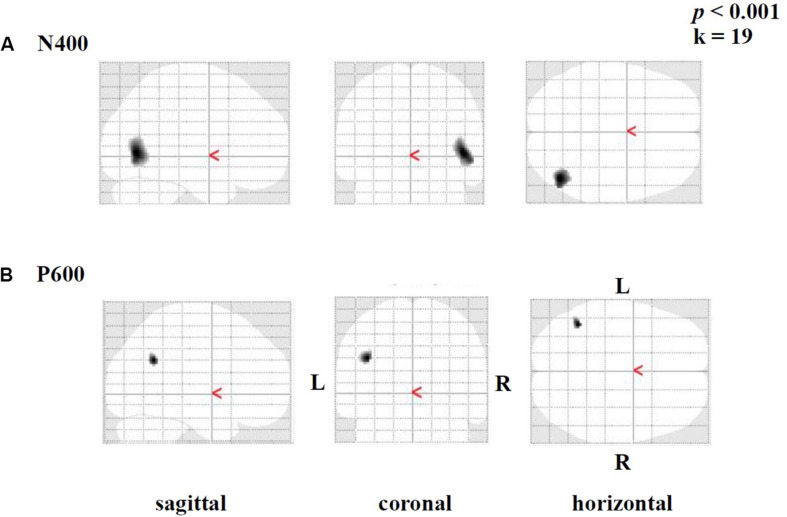
Statistical parametric maps (SPMs) reflecting correlations between relative increases of perfusion during the speech-discrimination task (semantic-anomaly paradigm) and the **(A)** N400 and the **(B)** P600 amplitudes, respectively. SPMs are shown as glass brain images. Note the predominant correlations in the temporal cortex (BA 37, **A**) and the parietal–occipital areas (in **B**).

Brain activation (speech condition minus rest condition) correlated (based on analyses without correction for multiple comparisons) in specific small brain regions negatively with the mean peak amplitude values of the N400 ERP (Figure 7A). An enhanced N400 ERP was related to a higher regional brain activation during the speech comprehension task in the following brain areas: the right temporal BA 37 and occipital in BA 19 (Figure 7A). Further correlations in smaller areas were seen in the left temporal (BA 37) and occipital areas (BA 17, 19; see Table 5). With regard to the P600 ERP, larger amplitudes were correlated (based on analyses without correction for multiple comparisons) with enhanced activation in the left parietal cortex (BA 39; Figure 7B). Additionally, smaller regions of positive correlation were observed in occipital regions (left and right BA 18 and left BA 19; see Table 6). None of the correlations performed with correction for multiple comparisons (FWE) revealed any suprathreshold voxels with *p* < 0.05.

**TABLE 5:** Results of negative correlations: difference image (speech condition - rest condition) vs. N400 EEG component (significance level used for inferences at a voxel level: *p* < 0.001, extent voxel threshold *k* = 0).

**Brain region**	**Corresponding Brodmann area**	**Hemisphere**	**x^a^**	**y^a^**	**z^a^**	**T**	**Nb voxel cluster**	**% cluster**
Temporal	BA 37	R	46	−62	2	5.6	364	9.6
Occipital	BA 19	R	46	−62	2	5.6	364	0.3
Temporal	BA 37	L	−40	−62	4	4.0	6	16.7
Occipital	BA 17	R	0	−92	2	4.0	4	25.0
Occipital	BA 19	L	−26	−74	22	3.9	1	100.0

**^*a*^MNI coordinates.**

**TABLE 6:** Results of positive correlations: difference image (speech condition - rest condition) vs. P600 EEG component (significance level used for inferences at a voxel level *p* < 0.001, extent voxel threshold *k* = 0).

**Brain region**	**Corresponding Brodmann area**	**Hemisphere**	**x^a^**	**y^a^**	**z^a^**	**T**	**Nb voxel cluster**	**% cluster**
Parietal	BA 39	L	−42	56	28	4.9	44	2.3
Occipital	BA 18	R	16	−66	−12	3.9	1	100.0
Occipital	BA 18	R	16	−72	−10	3.9	2	50.0
Occipital	BA 18	L	−16	−60	−10	3.9	2	50.0
Occipital	BA 19	L	−22	−86	−18	3.8	2	50.0

**^*a*^MNI coordinates.**

#### Audiometric and Cognitive Performance vs. EEG Components

Individual linear regression analyses between measures of audiometric/cognitive performance (Freiburg monosyllabic word test, HSM sentence test in quiet and in noise, GÖSA, verbal intelligence and fluency, working memory, and verbal learning) and EEG components (N1, P2, N400 and P600) were performed. None of these reached significance (*p* always > 0.05) and squares of the correlation coefficients were always below 0.15 excluding a relevant correlation between the respective data.

## Discussion

The present study aimed to better understand the high variability in CI outcomes. We used an innovative multimodal diagnostic approach, including brain-perfusion SPECT with tracer injection during EEG measurement to examine speech processing in CI users. Three main findings were obtained: First, the CI users activated a temporo-frontal network for speech processing and showed correlations between activation in the temporal gyrus and occipital regions on the one hand and cognitive ERP amplitudes on the other hand. This demonstrates a close connection between ERP effects and cortical activation in CI users. Second, the CI users with lower and higher speech comprehension showed different activation patterns for baseline brain activity (“rest condition”) as well as for activation during speech processing (“speech condition”), pointing to differential allocation of neural resources and strategies used for speech processing. Third, we observed strong correlations between the brain networks activated during speech processing and specific cognitive abilities, in particular working memory capacity and verbal memory functions, implying that these cognitive functions play a crucial role for speech comprehension in CI users.

### Brain Regions Recruited for Speech Processing in NH Listeners and in CI Users

The speech processing cascade, comprising the primary acoustic analysis, the identification of phonemes and words, and the integration of semantic and syntactic information, includes a complex system of interacting brain areas (Hickok and Poeppel, 2000; Friederici, 2002; Scott and Johnsrude, 2003). The traditional view of speech processing proposed the dominant involvement of the left-hemisphere inferior frontal and temporal cortices (Broca, 1861; Wernicke, 1874). However, more recent findings have suggested a bilateral involvement and the existence of two dorsal and two ventral pathways (Friederici et al., 2006; Saur et al., 2008; Saur et al., 2010; Friederici, 2012), with the dorsal streams connecting the superior temporal gyrus (STG) with the premotor cortex and BA 44, respectively, whereas the ventral streams connect, on the one hand, the STG to BA 45/47 and, on the other hand, the anterior temporal cortex to the frontal operculum. Regarding semantic sentence processing, temporal as well as inferior frontal cortical areas are involved (for a review, see, e.g., Friederici, 2002), in particular BA 45/47 and the left middle temporal gyrus (MTG). Further MEG and functional MRI (fMRI) findings with NH listeners support the essential role of the left MTG in semantic processing (see, e.g., Lau et al., 2008).

CI users have been shown to recruit similar brain regions and circuits for speech processing when compared with NH listeners, although they reveal lower activation in the temporal voice area (Giraud et al., 2000; Coez et al., 2008). Furthermore, the CI users show a compensatory increase in activation in the left inferior prefrontal cortex (Broca’s region) (Giraud and Truy, 2002), the anterior superior temporal phonologic region (Giraud et al., 2000), temporo-occipital visual areas (Giraud et al., 2001b; Giraud and Truy, 2002), parietal attentional regions (Giraud et al., 2000; Coez et al., 2014), and parahippocampal memory areas (Giraud et al., 2001c). Consistent with previous observations with CI users and NH listeners, in our study, the comparison of speech task versus rest revealed large activations in the bilateral temporal cortex (BA 41, 42, 22, 21, and 20) as well as the inferior prefrontal cortex (BA 45, 47) (Kutas and Hillyard, 1980a, b; Friederici, 2002; Van Petten and Luka, 2006; Hahne et al., 2012). Thus, our results are in line with previous observations by demonstrating the recruitment of a temporo-frontal network of brain areas during speech processing in CI users. Moreover, they reveal that a sentence comprehension task typically employed to study ERP effects is likewise suitable to achieve synchronously brain activations detectable in emission tomography.

Interestingly, we did not observe an activation of the inferior parietal cortex and the dorsal part of Broca’s area in the inferior prefrontal cortex (BA 44). This suggests that the dorsal pathway – known to be particularly involved in processing of syntactically complex sentences (Friederici, 2012) – was not considerably activated in our patients. This is plausible due to the fact that the sentences used in our discrimination paradigm were syntactically simple and their processing may have relied rather on the ventral than the dorsal pathways. Regarding the parietal areas, the lack of activation in these regions might be related to the fact that participants were stimulated *unilaterally*, while previous studies reporting parietal recruitment during speech processing are restricted to CI users with *bilateral* stimulation (Coez et al., 2014). However, our CI users showed supplementary activation in the hippocampus (BA 48), pointing to memory functions involved in performing the semantic-anomaly paradigm.

Interestingly, when correcting our data sets for the side of stimulation (left-sided stimulation flipped, right-sided stimulation unflipped), we did not observe activation in the ipsilateral primary auditory cortex (BA 41). Similarly, previous fMRI studies with NH listeners have reported that monaural presentation of speech results in a stronger contralateral activation of the primary auditory cortex (Jäncke et al., 2002; Stefanatos et al., 2008). Contralaterally predominant activation has also been observed with unilateral as opposed to bilateral stimulation in CI users (Green et al., 2011; Coez et al., 2014).

### Different Patterns of Brain Activity at Rest and Activation Related to Speech Processing in CI Users With Lower and Higher Performance

There is high interindividual variability in speech comprehension abilities across CI users (Lazard et al., 2012; Blamey et al., 2013). Differences between proficient and non-proficient CI users seem to exist already at the time before implantation, as indicated by the finding of distinct preimplantation activation patterns during a (written) word rhyming task between (prospective) lower and higher performance after implantation (Lazard et al., 2010). After implantation, proficient and non-proficient CI users have been shown to recruit the auditory cortex to a different degree, with reduced recruitment of the temporal voice area (with regard to extent and only unilaterally) in the poor performers when compared with good performers (Coez et al., 2008). However, in the present study, the activation of temporal regions (BA 20) was comparable between the two groups of CI users. This might be explained by the fact that the current study compared CI users with high and moderate speech comprehension (referred here as higher and lower CI performers), while the two aforementioned studies compared CI users with clearly different good and bad performance, leaving out a broad spectrum of patients with intermediate performance.

Despite the lack of a group difference in the temporal regions, we observed for the higher performing CI users additional activations of parietal and occipital regions, whereas for the group of lower performing subjects, we found a stronger activation of superior frontal areas. Similar to our results, previous studies have reported for good CI performers increased activity in temporo-occipital visual areas (Giraud et al., 2001b; Giraud and Truy, 2002) and parietal attentional regions (Giraud et al., 2000; Coez et al., 2014), suggesting compensatory networks of speech processing in these proficient individuals. The *auditor*y-evoked activation in the *visual* cortex might be related to functional, cross-modal reorganization of the *visua*l cortex that is used to compensate for the degraded auditory input via the CI. Accordingly, it has been shown in previous studies that activation of the *visual* cortex by *auditor*y stimulation is positively related to the CI performance (Giraud et al., 2001b, c; Strelnikov et al., 2013; Chen et al., 2016). Thus, it can be speculated that, in the present study, the two groups of CI users used different compensatory strategies that may be related to differences in cross-modal reorganization of the visual and auditory cortex. CI speech performance seems to be good as long as the (beneficial) *auditor*y-evoked activation in the *visual* cortex is higher than the (maladaptive) *visual*-evoked activation in the *auditory* cortex (Chen et al., 2016). This is in line with the current findings, showing that specifically the higher performers showed an enhanced beneficial cross-modal reorganization in the *visual* cortex.

Regarding the lower performing CI users, we observed increased superior frontal activations (BA 9) compared with those with higher performance. This is in contrast to a previous study reporting that activation in non-proficient CI users is restricted to temporal areas (Mortensen et al., 2006). The discrepancy of results is likely attributable to variations in methodology, in particular in terms of the experimental task (active vs. passive task) and the speech comprehension of the lower performing group (open-set speech comprehension: ≤ 60 vs. ≥ 65%). Furthermore, our observation of increased superior frontal activation particularly in the poorer performing CI users is meaningful, as it might reflect enhanced neural resource allocation due to limitations in electrical hearing. Indeed, peripheral factors, for instance the distance of the CI electrode arrays to the modiolar wall and the number of surviving spiral ganglion cells have been shown to affect speech comprehension with the CI (Nadol, 1997; Holden et al., 2013). In case of suboptimal peripheral conditions, the resulting strong(er) mismatch between the CI input and the attributes stored in the long-term memory may require additional explicit processing and involve cognitive resources, in particular working memory functions (Rönnberg et al., 2013; Finke et al., 2015), and may cause enhanced listening effort (Berding et al., 2015). Indeed, it has been shown previously that NH listeners recruit additional prefrontal regions specifically in difficult listening conditions in which the listening effort is enhanced (Davis and Johnsrude, 2003; Peelle, 2018). Thus, it is likely that during speech processing, the lower CI performers rely on a different processing strategy compared with the higher performers by particularly allocating executive cognitive resources located in specific frontal regions. However, this compensatory strategy seems to be limited, as indicated by the fact that the lower CI performers did not reach the speech comprehension performance levels of higher CI performers.

Additionally, we observed CI-outcome-related distinct brain activity patterns at rest: lower performers showed higher resting state perfusion in motor cortex and parietal areas, whereas higher performers showed higher perfusion in (inferior) frontal (BA47), inferior temporal (BA20), temporopolar (BA38), and hippocampal (memory) regions. The prognostic relevance of resting activity has been demonstrated before (Lee et al., 2007; Giraud et al., 2011; Strelnikov et al., 2015a; Suh et al., 2015). It has been shown, for example, that a low (resting-state) activity in the primary auditory/superior temporal cortex – indicating that no maladaptive cross-modal visual take-over has taken place – is related to a better CI outcome (Lee et al., 2007; Strelnikov et al., 2015a; Suh et al., 2015). The same has been observed for an increased activity in the prefrontal cortex, in particular Broca’s area (Lee et al., 2007; Suh et al., 2015). Our results are consistent with these previous findings by showing that CI users with higher performance have increased resting-state perfusion particularly in left-sided (inferior) frontal (BA 11, 47, 25) areas.

Overall, our results show that CI users with lower and higher speech comprehension recruit distinct brain networks not only during speech processing but also during rest. This points to different compensation strategies for the processing of the degraded CI speech signal and suggests different adaptation of the brain in response to the individual auditory experience.

### Cognitive Abilities and Their Relationship With Brain Activation During Speech Processing

The performance of CI users has been shown to be influenced by cognitive factors, like verbal fluency and working memory capacity (Rönnberg et al., 2013; Finke et al., 2016a). However, it is currently widely unknown how individual differences in cognitive abilities relate to cortical response patterns during speech processing. The present study suggests that enhanced activation in (predominantly) temporo-frontal areas during speech processing is positively associated with higher word recognition scores and higher verbal intelligence. Temporo-frontal regions encompass areas related to auditory, executive, and memory functions that are used for speech comprehension in the context of the semantic-anomaly paradigm (Lau et al., 2008; Kutas and Federmeier, 2011). Consistent with our observations, a coupling between speech recognition scores and activation of auditory areas, in particular the Heschl’s gyrus (BA 41), the superior temporal, and the angular gyrus, has been observed previously (Łukaszewicz-Moszyńska et al., 2014). In sum, these results underpin that cognitive abilities and speech comprehension ability in CI users relate to specific speech-evoked cortical activation in temporo-frontal regions. Thus, enhanced activation in these regions seems to allow better verbal abilities and higher word comprehension ability with a CI.

Additionally, we observed better verbal abilities and enhanced working memory capacity being associated with increased activations in the superior and middle temporal gyrus. This indicates that better cognition results in enriched activations of typical auditory areas, which might be related to better speech comprehension with CI as well. As all cognitive tests recall phonetic, vocabulary, and memory abilities simultaneously, this might indicate the use of similar resources during the performance of these cognitive tests and the semantic speech task.

### ERPs and Their Relationship With Brain Activation Detected With SPECT

In the current study, the CI users showed an N1-P2 ERP in response to the onset of the sentence, indicating processing of speech at the level of the auditory cortex in CI users (Naatanen and Picton, 1987; Friesen and Picton, 2010). Furthermore, the difference waves (ERP_critViol_ - ERP_critCorr_), time locked to the onset of the final word of the sentence, revealed a more negative amplitude to sentences with a semantic violation compared to correct sentences, referred to as an N400 effect (Kutas and Hillyard, 1980c; Kutas and Hillyard, 1984). The N400 is considered as an index of neural effort of automatic word-into-context integration (Strauss et al., 2013). In other words, it is assumed to reflect the level of difficulty with which a word is integrated in the respective context (Van Petten et al., 1999). Interestingly, the observed latency of the N400 effect in CI users (∼700–800 ms) was delayed when compared with the N400 latency of NH listeners reported in the literature (∼400 ms; e.g., Lau et al., 2008). Nevertheless, it was comparable to a previous study, reporting a delayed N400 response in CI users when compared with NH listeners (Hahne et al., 2012). These results suggest that adverse listening conditions, as experienced by CI users with the degraded auditory input from the implant, lead to more effortful and thus delayed semantic integration processes (Finke et al., 2016a).

The present study showed negative correlations between the N400 response and the temporal (in particular BA 37), as well as activation in the visual cortex (SPECT difference image: speech condition - rest condition). Specifically, more negative N400 amplitudes in the present study were found to be associated with higher perfusion in a broad network, including temporal and occipital regions. The N400 has been suggested to be primarily generated in the left middle temporal gyrus (Lau et al., 2008; Friederici, 2012). BA 37 has also been suggested previously to be part of the semantic processing network (Lau et al., 2008; Ardila et al., 2015). The observed correlation with the temporal region suggests this region to be as well involved in the generation of the N400 in CI users. However, results of the present study also showed a strong correlation with visual areas, strongly suggesting an additional cross-modal recruitment of visual areas during semantic processing in CI users. The engagement of occipital areas might be related to the fact that although sentences are presented purely auditorily, they might be internally visualized. Furthermore, it has been previously reported that CI users show an enhanced audiovisual coupling (Schierholz et al., 2015, 2017; Strelnikov et al., 2015b) and that activation of the *visual* cortex by *auditor*y stimulation is positively related to the CI performance (Giraud et al., 2001b, c; Strelnikov et al., 2013; Chen et al., 2016), indicating that cross-modal reorganization in the visual cortex and enhanced audiovisual coupling support speech processing in CI users.

The difference waves revealed that the N400 was followed by a positive deflection at around 900 ms after the final word onset. This late component has been referred to the P600 (Osterhout and Holcomb, 1992), which typically peaks between 300 and 800 ms (Friederici et al., 2000; Friederici, 2006). Similar to the N400 response, our results suggest a delayed P600 response in our CI users, which can be attributed to the degraded input from the implant, leading to delayed higher-level speech processing. Traditionally, the P600 has been related to syntactic processing effort in general and it is, for example, observed in the context of syntactical repair, syntactical complexity, and difficulties with syntactic integration (Osterhout and Holcomb, 1992; Hagoort et al., 1993; Kaan et al., 2000; Friederici et al., 2002). Nevertheless, the P600 has been recently discussed in a semantic context as well (see, e.g., Bornkessel-Schlesewsky and Schlesewsky, 2008). The studies by Kolk et al. (2003) and van Herten et al. (2005), for example, observed a P600 effect elicited by semantic anomalies, challenging the merely syntactic account of the P600. This view has been supported by the study by Shen et al. (2016), suggesting that the P600 reflects a general mechanism of semantic reinterpretation and conflict monitoring that leads to the retrieval of word knowledge from long-term memory. A systematic review on the effects of semantic incongruency by Van Petten and Luka (2012) has identified 21 out of 64 studies that exhibited a biphasic N400/P600 effect to incongruent sentences, confirming that indeed the P600 is elicited by semantic anomalies in sentences. Regarding the generators of the P600, the bilateral medial/posterior temporal cortex has been identified in NH listeners (Service et al., 2007). The current results showed that stronger P600 amplitudes were associated with higher perfusion in parietal and occipital areas. The parietal correlation involved BA 39 (angular gyrus), which has been shown to be a part of the semantic processing network (Lau et al., 2008). Additionally, stronger P600 responses were associated with higher perfusion in occipital areas, suggesting once again that CI users recruit additional visual regions during semantic processing.

Our results revealed that both the N400 and the P600 ERPs correlate with the activation of a broad but distinct network. Importantly, both components correlate with activation in occipital areas (N400: BAs 17, 19; P600: BAs 18, 19). This extends previous research by showing that CI users strongly rely on visual cortex activation during semantic speech processing. All in all, our findings show a close connection between ERP effects and cortical activation in CI users, demonstrating that the combination of SPECT and EEG measurements provides unique and valuable insights into the cognitive processes underlying speech comprehension in CI users.

Our results also extend previous studies by indicating that not only sensory but also cognitive ERPs, in particular the N400 and the P600 response, can distinguish – although in the present study not with statistical significance, but at least on the descriptive level – between CI users who have higher versus lower speech comprehension. Thus, our results might point to potential different abilities of lower and higher performing CI users in detecting and integrating semantic violations in sentences. We speculate that increasing the sample size would have resulted in less variance in the data and statistical group differences for the N400 and the P600 amplitudes, respectively. Interestingly, on the descriptive level, our results showed that the amplitudes of the N1, P2, and N400 are *reduced* in the lower compared to the higher CI performers, while we observed on a descriptive level the opposite pattern, that is an *enhanced* amplitude, for the P600 in the lower compared to the higher performers. These descriptive observations support the significant group differences revealed in the SPECT data and point together to different strategies for speech comprehension in lower and higher performers. While lower performers invest less neural resources in automatic word-into-context integration (reflected by the reduced N400 response, here observed on a descriptive level), they use additional explicit processing resources for semantic reinterpretation and retrieval of word knowledge from the long-term memory (reflected by the enhanced P600 response, here observed on a descriptive level). Furthermore, this is in line with our observation of increased frontal activation in the lower compared to the higher CI performers and the Ease of Language Understanding (ELU) model (Rönnberg et al., 2013), according to which additional cognitive resources are required for speech comprehension in demanding listening situations, which particularly applies to CI users with lower speech comprehension.

## Conclusion

The present study showed that higher and lower CI outcome is associated with different brain activation patterns. Furthermore, our results revealed the meaningful applicability of a combined EEG and SPECT multimodal diagnostic approach for examination of speech processing in CI users. Our results revealed that based on a sentence discrimination task, activation of a temporo-frontal network can be detected in both diagnostic modalities correspondingly to previous observations with PET in CI users. Furthermore, the present results revealed significantly different activation patterns between lower and higher CI performers. The results point to the use of different compensational strategies for the degraded auditory input and different adaptations of the brain in response to the individual auditory experience for groups of CI users with higher and lower performance. Moreover, differences between these groups of CI users, at least on the descriptive level, were observed for the EEG data. Here, the lower performers showed reduced amplitudes for the sensory ERPs (auditory N1 and P2) and the later cognitive N400 ERP, whereas the opposite pattern, that is enhanced amplitudes, were observed for the P600 ERP in the lower compared to the higher performers. These findings point to more pronounced deficits/limitations in CI users with lower performance due to a particularly degraded auditory input and compensatory strategies to overcome these limitations by a stronger recruitment of higher-cognitive resources, involving frontal regions.

## Data Availability Statement

The raw data supporting the conclusions of this article will be made available by the authors, without undue reservation.

## Ethics Statement

The studies involving human participants were reviewed and approved by the local ethics committee at Hannover Medical School (vote no. 6678) and the German Federal Office for Radiation Protection (reference number Z 5-22461/2-2014-012). The patients/participants provided their written informed consent to participate in this study.

## Author Contributions

MK designed the study, recruited the study sample, collected and analyzed EEG and SPECT data, wrote the manuscript, and prepared the tables and figures. IS designed the study, recruited the study sample, collected and analyzed EEG data, contributed to the writing of the manuscript, and prepared the figures. MM contributed to the design of the study, assisted during the data collection, provided technical support, and revised the manuscript. FW provided technical support for the SPECT measurement, contributed to the SPECT data analysis, and revised the manuscript. AH contributed to the experimental setup, advised on the EEG data analysis, and revised the manuscript. AB contributed to the setup of the EEG facility. LG provided expertise for the SPECT acquisition protocol. FB allocated personnel resources enabling SPECT acquisitions. PS conceived and designed the study, supervised the EEG data collection, contributed to the EEG data analysis, and contributed to the writing of the manuscript. GB conceived and designed the study, supervised the SPECT data acquisition, contributed to the SPECT data analysis, and contributed to the writing of the manuscript. All authors contributed to the article and approved the submitted version.

## Conflict of Interest

The authors declare that the research was conducted in the absence of any commercial or financial relationships that could be construed as a potential conflict of interest.

## Footnotes

1 https://www.memoryclinic.ch/de/main-navigation/neuropsychologen/cerad-plus/

2 http://www.billauer.co.il/peakdet.html
